# Supplementary material for: Primary kidney disease modifies the effect of comorbidities on kidney replacement therapy patients’ survival
Source: PLoS One. 2021 Aug 20;16(8):e0256522. doi: 10.1371/journal.pone.0256522 (PMC8378722; doi:10.1371/journal.pone.0256522)
Supplement: S1 Table — (DOCX) [file pone.0256522.s001.docx]

**SUPPLEMENTARY MATERIAL**

**S1 Table.** **Unadjusted effect of comorbidities on relative risk of death according to primary kidney disease**

| **Comorbidity,**  **RR (95%CI)** | **Type 2 diabetes** | **Type 1 diabetes** | **Glomerulo-nephritis** | **Polycystic kidney disease** | **Nephro-sclerosis** | **Other or unknown diagnoses** | **All patients** | **Interaction P value^a^** |
| --- | --- | --- | --- | --- | --- | --- | --- | --- |
| **Coronary artery disease** | 1.67 (1.49–1.87) | 2.10 (1.77–2.50) | 3.57  (2.88–4.43) | 4.05 (2.97–5.51) | 2.42 (1.94–3.03) | 2.10 (1.89–2.33) | 2.47 (2.32–2.63) | <0.001 |
| **Peripheral vascular disease** | 1.98 (1.75–2.23) | 2.64 (2.21–3.16) | 3.47  (2.48–4.86) | 2.02 (1.26–3.23) | 2.23 (1.76–2.82) | 2.01 (1.75–2.31) | 2.51 (2.34–2.70) | 0.002 |
| **Left ventricular hypertrophy** | 1.14 (1.01–1.28) | 1.32 (1.11–1.58) | 1.72  (1.39–2.12) | 1.38 (1.02–1.88) | 1.03 (0.82–1.30) | 1.66 (1.49–1.84) | 1.56 (1.47–1.66) | <0.001 |
| **Cerebrovascular disease** | 1.43 (1.24–1.65) | 2.09 (1.68–2.61) | 2.54  (1.91–3.37) | 1.20 (0.79–1.82) | 1.77 (1.33–2.37) | 1.90 (1.64–2.21) | 1.83 (1.68–1.99) | <0.001 |
| **Heart failure** | 1.91 (1.66–2.19) | 3.48 (2.69–4.51) | 6.35  (4.62–8.74) | 6.61 (3.57–12.22) | 2.22 (1.67–2.94) | 2.75 (2.39–3.15) | 3.21 (2.96–3.49) | <0.001 |
| **Malignancy** | 1.14 (0.95–1.38) | 1.94 (1.31–2.87) | 2.87  (2.19–3.78) | 3.20 (2.19–4.68) | 1.19 (0.89–1.59) | 1.61 (1.44–1.79) | 1.80 (1.66–1.94) | <0.001 |
| **Normal weight**  **(BMI 20–30 kg/m^2^)** | 1 | 1 | 1 | 1 | 1 | 1 | 1 | 0.001 |
| **Obesity**  **(BMI > 30 kg/m^2^)** | 0.91 (0.81–1.02) | 1.05 (0.84–1.31) | 1.59  (1.27–1.97) | 1.24 (0.88–1.75) | 0.80 (0.60–1.08) | 0.98 (0.86–1.11) | 1.25 (1.16–1.33) |  |
| **Underweight**  **(BMI < 20 kg/m^2^)** | 1.22 (0.80–1.85) | 1.61 (1.19–2.18) | 0.94  (0.63–1.40) | 1.47 (0.84–2.59) | 1.09 (0.66–1.81) | 1.20 (1.03–1.40) | 1.21 (1.08–1.37) |  |
| **Systolic blood pressure**  **> 140 mmHg** | 0.66 (0.58–0.74) | 0.84 (0.70–1.01) | 0.85  (0.70–1.04) | 1.18 (0.90–1.54) | 1.13 (0.89–1.44) | 0.91 (083–1.00) | 0.89 (0.84–0.94) | <0.001 |
| **Diastolic blood pressure**  **> 90 mmHg** | 0.67 (0.57–0.78) | 0.71 (0.59–0.84) | 0.43  (0.35–0.54) | 0.78 (0.58–1.03) | 0.61 (0.46–0.80) | 0.65 (0.57–0.73) | 0.56 (0.52–0.60) | 0.008 |

^a^Interaction between diagnosis group and comorbidity

RR, relative risk of death; 95% CI, 95% confidence interval; BMI, body mass index
