# Supplementary material for: Primary kidney disease modifies the effect of comorbidities on kidney replacement therapy patients’ survival
Source: PLoS One. 2021 Aug 20;16(8):e0256522. doi: 10.1371/journal.pone.0256522 (PMC8378722; doi:10.1371/journal.pone.0256522)
Supplement: S2 Table — (DOCX) [file pone.0256522.s002.docx]

**S2 Table.** **Age- and sex-adjusted effect of comorbidities on relative risk of death according to primary kidney disease**

| **Comorbidity,**  **RR (95%CI)** | **Type 2 diabetes** | **Type 1 diabetes** | **Glomerulo-nephritis** | **Polycystic kidney disease** | **Nephro-sclerosis** | **Other or unknown diagnoses** | **All patients** | **Interaction P value^a^** |
| --- | --- | --- | --- | --- | --- | --- | --- | --- |
| **Coronary artery disease** | 1.44 (1.29–1.62) | 1.46 (1.22–1.76) | 1.71 (1.36–2.14) | 2.29 (1.65–3.17) | 1.68 (1.33–2.12) | 1.40 (1.26–1.56) | 1.68 (1.58–1.79) | <0.001 |
| **Peripheral vascular disease** | 1.94 (1.72–2.19) | 1.98 (1.64–2.38) | 1.99 (1.42–2.80) | 1.82 (1.14–2.91) | 1.81 (1.43–2.30) | 1.49 (1.29–1.71) | 2.04 (1.90–2.20) | 0.098 |
| **Left ventricular hypertrophy** | 1.12 (1.00–1.27) | 1.21 (1.01–1.45) | 1.38 (1.11–1.71) | 1.18 (0.86–1.62) | 1.34 (1.06–1.70) | 1.27 (1.15–1.42) | 1.34 (1.26–1.43) | 0.365 |
| **Cerebrovascular disease** | 1.34 (1.16–1.55) | 1.51 (1.21–1.90) | 1.88 (1.41–2.50) | 1.19 (0.79–1.81) | 1.59 (1.19–2.13) | 1.40 (1.21–1.63) | 1.50 (1.38–1.63) | 0.167 |
| **Heart failure** | 1.74 (1.52–2.01) | 2.35 (1.80–3.07) | 4.76 (3.44–6.57) | 4.97 (2.67–9.24) | 2.06 (1.55–2.73) | 2.23 (1.94–2.56) | 2.43 (2.24–2.64) | <0.001 |
| **Malignancy** | 0.92 (0.76–1.11) | 1.29 (0.86–1.93) | 1.49 (1.13–1.97) | 1.99 (1.35–2.92) | 0.85 (0.68–1.09) | 1.12 (1.00–1.25) | 1.11 (1.02–1.20) | <0.001 |
| **Normal weight**  **(BMI 20–30 kg/m^2^)** | 1 | 1 | 1 | 1 | 1 | 1 | 1 | 0.008 |
| **Obesity**  **(BMI > 30 kg/m^2^)** | 0.94 (0.84–1.06) | 0.96 (0.77–1.20) | 1.77 (1.42–2.21) | 1.19 (0.84–1.68) | 0.97 (0.72–1.30) | 1.01 (0.89–1.15) | 1.21 (1.13–1.29) |  |
| **Underweight**  **(BMI < 20 kg/m^2^)** | 1.25 (0.82–1.89) | 1.79 (1.32–2.42) | 1.42 (0.95–2.13) | 1.66 (0.94–2.03) | 1.96 (1.16–3.30) | 1.52 (1.30–1.78) | 1.50 (1.33–1.69) |  |
| **Systolic blood pressure**  **> 140 mmHg** | 0.67 (0.59–0.76) | 0.82 (0.68–0.98) | 0.82 (0.67–1.00) | 1.10 (0.84–1.45) | 0.98 (0.77–1.25) | 0.85 (0.78–0.94) | 0.89 (0.84–0.94) | 0.019 |
| **Diastolic blood pressure**  **> 90 mmHg** | 0.79 (0.67–0.93) | 1.05 (0.86–1.27) | 0.85 (0.68–1.07) | 1.01 (0.76–1.35) | 0.93 (0.71–1.24) | 1.00 (0.88–1.14) | 0.85 (0.78–0.91) | 0.049 |

^a^Interaction between diagnosis group and comorbidity

RR, relative risk of death; 95% CI, 95% confidence interval; BMI, body mass index
