# Supplementary material for: Primary kidney disease modifies the effect of comorbidities on kidney replacement therapy patients’ survival
Source: PLoS One. 2021 Aug 20;16(8):e0256522. doi: 10.1371/journal.pone.0256522 (PMC8378722; doi:10.1371/journal.pone.0256522)
Supplement: S3 Table — (DOCX) [file pone.0256522.s003.docx]

**S3 Table.** **Age- and sex-adjusted relative risk of death according to the number of comorbidities in various groups of primary kidney disease.**

|  | **Type 2 diabetes** | **Type 1 diabetes** | **Glomerulo-nephritis** | **Polycystic kidney disease** | **Nephro-sclerosis** | **Other or unknown diagnoses** | **All patients** |
| --- | --- | --- | --- | --- | --- | --- | --- |
| **No comorbidity** | 1 | 1 | 1 | 1 | 1 | 1 | 1 |
| **One comorbidity**  **RR (95% CI)** | 1.31  (1.11–1.53) | 1.27  (1.04–1.56) | 1.61  (1.28–2.03) | 1.34  (0.98–1.81) | 1.14  (0.82–1.60) | 1.40  (1.25–1.58) | 1.50  (1.39–1.62) |
| **Two comorbidities**  **RR (95% CI)** | 1.57  (1.33–1.86) | 1.83  (1.45–2.32) | 2.02  (1.51–2.70) | 2.17  (1.48–3.18) | 1.96  (1.39–2.77) | 1.87  (1.62–2.16) | 2.11  (1.94–2.30) |
| **Three or more comorbidities**  **RR (95% CI)** | 2.27  (1.91–2.69) | 2.63  (2.02–3.43) | 4.62  (3.29–6.49) | 4.36  (2.48–7.68) | 2.35  (1.68–3.30) | 2.25  (1.92–2.63) | 2.96  (2.70–3.25) |

RR, relative risk of death; 95% CI, 95% confidence interval
