# Supplementary material for: Primary kidney disease modifies the effect of comorbidities on kidney replacement therapy patients’ survival
Source: PLoS One. 2021 Aug 20;16(8):e0256522. doi: 10.1371/journal.pone.0256522 (PMC8378722; doi:10.1371/journal.pone.0256522)
Supplement: Attachment — Submitted filename: Response to Reviewers PLOS ONE.docx [file pone.0256522.s004.docx]

**The authors’ detailed response to the Additional Editors and Reviewers comments on the manuscript “Primary kidney disease modifies the effect of comorbidities on kidney replacement therapy patients’ survival”**

Additional Editor Comments:

Manuscript is of interest in the Nephrology community. It is a large cohort of patients in Finland looking at the effect of comorbidities by primary kidney disease modifying survival in patients on dialysis.

Also, please modify "renal" to "kidney" throughout the manuscript, whenever it is possible to refer to kidney disease or kidney function, as it is the new terminology recommendation by the KIDGO and to make it more patient-centered.

*Authors’ reply: Thank you for this notice. We have now modified the manuscript and replaced “renal” to “kidney”.*

In the ethics statement in the manuscript and in the online submission form, please provide additional information about the patient records/samples used in your retrospective study, including the date range (month and year) during which patients' medical records/samples were accessed.

*Authors’ reply: We have added this more detailed information about patient records to the Materials and methods (page 5, lines 79–81 and page 6, lines 85–86).*

We note that you have indicated that data from this study are available upon request. PLOS only allows data to be available upon request if there are legal or ethical restrictions on sharing data publicly.

*Authors’ reply: Our data are retrieved from the patient-level data and the sharing is restricted by the EU General Data Protection Regulation 2016/679.*

Reviewer #1: 
This manuscript is well written with a few minor revisions. The authors were able to address the issue on whether primary renal disease modifies the effect of comorbidities on the survival of patients on chronic dialysis therapy. If published, I think this will guide clinicians make management decisions especially on how often they need to follow up with a patient. This will also help in prognostication as their data showed that patients with glomerulonephritis and polycystic kidney disease have higher risk of mortality if they have coronary artery disease or heart failure compared to other patients.

I do have some points that I would like some clarification or need some revision.
1. Their definition of overweight was a BMI > 30. Based on current criteria by the WHO, being overweight is defined as a BMI of 25.0 to <30. It might be better if they change the term overweight to obesity instead.

*Authors’ reply: Thank you for this comment. We agree that obesity is better term for BMI > 30 and we have now changed this in the manuscript.*

2. Their definition of underweight was BMI <20. Based on current criteria by the WHO, being underweight is defined as a BMI of < 18.5. However, I acknowledge that this might potentially impact their results if they adjust the cut-off. My concern is that it may misinform the readers if they used different cut-off criteria.

*Authors’ reply: We acknowledge this potential misinformation and we have now clarified this in the Methods (page 5, lines 68–69).*

3. I would like to know the rationale for not including hypertension as one of the 6 comorbidities. As I compared the data from Table 2 and Table 3, it appears that they looked at patients who had elevated blood pressures and patients who were on anti-hypertensive medications. The total proportion of patients on medication was 88.9% while those with high blood pressure was 65.7% for those with elevated SBP and 24.3% for those with elevated DBP. It would be helpful for the readers to know if patients who were diagnosed with hypertension, whether controlled or not, and whether on medication or not had any significant difference in mortality.

*Authors’ reply: We did not include hypertension as one of the comorbidities because it is more a risk factor for most of these other comorbidities than an individual comorbidity. The results were similar whether or not patients were on antihypertensive medication. We have added a comment on this in the Results (page 10, line 143).*

4. The authors need to revise the language especially in the discussion part to improve readability. The specific part that needs revision is found on line 180-181, line 209-210.

*Authors’ reply: We have now rephrased these sentences and specifically gone through the Discussion and made some changes to improve readability. We hope you find this acceptable, but we are willing to make any further changes as needed.*

Reviewer #2: The authors have described "Primary renal disease modifies the effect of comorbidities on renal replacement therapy patients’ survival". After reading the manuscript thoroughly, i suggest following modifications.

1) Authors include good cohort of RRT patients and studied retrospectively. Can authors kindly explain if its done outpatient setting only or have included patients with continuous renal replacement therapies while in hospital too.

*Authors’ reply: Thank you for this clarification. Only patients on chronic kidney replacement therapy and in outpatient setting were included. We have added a sentence on this issue in the Materials and methods (page 4, line 63).*

2) Can authors mention if only hemodialysis patients were included / or patients on home therapies were included too as that could change the effect of co morbidities and primary disease on mortality.

*Authors’ reply: Also patients on home hemodialysis and peritoneal dialysis were included. We have added this information on other treatment modalities at the initiation of kidney replacement therapy in the Results (page 7, lines 109–110).*

3) Table-1 indicates that among pts with polycystic kidney disease, significant number 9.6 % underwent renal transplant as compared to type 2 DM with 3.1%. Can authors elaborate if that has any potential impact on over all mortality .

*Authors’ reply: It is true that kidney transplantation is associated with better survival and may well have an impact on overall mortality but the causes (comorbidities and other factors) in patients who did not receive kidney transplant also affect mortality. Survival is better in patients with PKD than in patients with type 2 diabetes, they have fever comorbidities, and they receive more kidney transplants. However, the association between comorbidities and mortality is stronger in patients with PKD than in patients with type 2 diabetes. Therefore, the difference between the number of kidney transplants in these groups should not explain our results. In addition, the criteria for accepting patients on transplantation waitlist are the same regardless of the primary kidney disease.*

4) Table-2 indicates that most of the co morbidities including CAD, peripheral vascular disease, left ventricular hypertrophy, more than 3 Co morbidities is highest among Type 2 DM and patients with Nephrosclerosis, which in turn correlate with increased mortality. However in the results sections, authors concluded that Polycystic kidney disease, GN are associated with increase mortality. How do authors explain this variation.

*Authors’ reply: As you mentioned, the patients with type 2 diabetes or nephrosclerosis have the highest number of comorbidities and the worst survival. The patients with polycystic kidney disease or glomerulonephritis have better prognosis, but in this study we show that comorbidities have a stronger worsening effect on survival among these patients. Therefore, if patients have multiple comorbidities the difference in survival is smaller between the primary kidney disease groups.*

5)Multiple studies mentioned uncontrolled hypertension as independent risk of mortality among ESRD patients on RRT. However the results are the study are opposite. Can authors elaborate on this

*Authors’ reply: It is true that treatment of hypertension reduces mortality in patients on dialysis, but also other epidemiological studies than ours have shown reverse association with hypertension and mortality among RRT patients. This may be due to increased mortality associated with hypotension. We have elaborated this issue now in the Discussion and added two references (page 15, lines 234–237).*

6) Appears that majority on patients in Finland are white population and hence these results could not be generalized to other parts of world with predominant black / asian population. Would be interesting to see sub group analysis on older age and race if they preset with similar findings.

*Authors’ reply: Unfortunately, we do not have the information on race. Even if we would have this information it would be difficult to present reliable results according to race because these subgroups would be too small. Also analyses according to age groups would reduce the number of patients and events too much in some diagnosis groups. For example, there were only 13 patients with type 1 diabetes over the age of 75 and 72 patients with nephrosclerosis under the age of 55. There were similar trends between diagnosis groups and number of comorbidities in different age groups as in our main results, but due to this insufficient number of patients in various subgroups, we do not think these results would be representative of this manuscript, but we can add these as needed.*
