# Supplementary material for: Primary kidney disease modifies the effect of comorbidities on kidney replacement therapy patients’ survival
Source: PLoS One. 2021 Aug 20;16(8):e0256522. doi: 10.1371/journal.pone.0256522 (PMC8378722; doi:10.1371/journal.pone.0256522)
Supplement: Attachment — Submitted filename: Response to reviewers.docx [file pone.0256522.s005.docx]

**The authors’ detailed response to the Additional Editors and Reviewers comments on the manuscript “Primary kidney disease modifies the effect of comorbidities on kidney replacement therapy patients’ survival”**

Additional Editor Comments:

Thank you for submitting this revised version of your manuscript and addressing our reviewers comments. Please address new comments made by our reviewers, specifically review data in supplemental tables, and body of the manuscript were you are mention a higher risk of mortality in patients with PKD and GN when compared to the other subsets., while in the text you state that their survival rate is similar to other patients with multiple comorbidities.

*Authors’ reply:*

*In the manuscript, we show that the effect of comorbidities on the risk of death is different in different kidney disease diagnoses, and in PKD and GN patients the effect is the strongest. This results in that the survival prognosis of PKD and GN patients with multiple comorbidities is similar to that of patients in other diagnostic groups with multiple comorbidities, although the prognosis is better in PKD and GN patients without comorbidities than in patients with other kidney disease diagnoses without comorbidities. We have worked to improve the manuscript to make this message clearer. Please also see the response to these comments of the Reviewers.*

Reviewer #1:

I have reviewed the revised version of your manuscript thoroughly. Thank you for responding to my comments and clarifying the information. I am satisfied with your revisions for the cutoff scores for the obesity and underweight. I am also satisfied with your explanation why hypertension was not included as a comorbidity. It was also helpful that you mentioned that patients with higher blood pressure had lower risk of mortality which further supports the notion that intradialytic hypotension is worse for patients on KRT.
After going over the manuscript and the data, I do have some additional comments. My main take away after reading is that I agree that we must take the primary renal disease into account and not just the comorbidities of the patient when thinking about prognosis. But I think there needs to be more emphasis as to why patients with PKD or GN who have underlying heart failure, malignancy or coronary artery disease are associated with higher mortality rates compared to the other subset of patients.
• The data is clear that patients with PKD or GN have less comorbidities (Table 2) and better survival (Table 1) compared to those with diabetes or nephrosclerosis. But then both the adjusted and unadjusted data show that they have a higher risk of mortality if they have heart failure, malignancy or coronary artery disease. It might be helpful to explain why patients with PKD or GN still have better survival compared to the other subsets despite having a higher risk of mortality when they have specific comorbidities.

*Authors’ reply:*

*Thank you for this comment because this is an essential issue for readers to understand correctly. Patients with PKD or GN without comorbidity have the lowest risk of mortality compared to the patients with other primary kidney disease without comorbidities. When PKD or GN patients have comorbidities the relative risk of death compared to PKD or GN patients without comorbidities increases more than it increases in patients with other kidney diseases when assessing the effect of comorbidities on mortality in that diagnosis group. This results a bigger difference between the survival probability of PKD or GN patients with and without comorbidities than in other primary kidney disease groups. Therefore, the survival is similar in all diagnosis groups when patients with multiple comorbidities are compared. We have clarified this issue in the Results, page 9, line 130, and page 12, line 170, and in the Discussion, page 12, lines 180 and 186.*

• Since patients with PKD or GN had higher rates of kidney transplantation and may have an impact on survival, were you able to see if the results will be affected after adjusting for kidney transplantation? If you think this is not worthwhile to look at, then maybe it will be helpful to provide a brief explanation in the manuscript as to why you think it will not impact the results of your study.

*Authors’ reply:*

*It is likely that kidney transplantation has an effect on survival and patients with PKD or GN had more often transplantation. Therefore, we made a sensitivity analysis where patients were censored at kidney transplantation and the results were similar. This is mentioned in page 11, line 154. We think that this is a better way to take an impact of kidney transplantation into account than adjustment for kidney transplantation, because in that case we would adjust the estimate of patients survival probability at the start of kidney replacement therapy with a future event (transplantation). However, we analysed how number of comorbidities associated with risk of death and multivariable model on comorbidities’ association with the risk of death with adjustment also for kidney transplantation and the results were similar, but due to the reasons mentioned above we did not add this information in the manuscript.*

• After looking at the data on Table S3, it appears that patients who have PKD or GN with 3 or more comorbidities have a higher risk of death compared to the other subsets. But then, in the discussion, it mentioned that these patients have similar survival compared to other patients (line 198-199). Is there an explanation for this variation?

*Authors’ reply:
Table S3 compares the relative risk of death according to number of comorbidities within that one kidney diagnosis group. PKD patients without comorbidity have median survival time over 18 years which is higher than in other diagnosis groups, but the relative risk of death associated with number of comorbidities is greater than in PKD patients than in other diagnosis groups and therefore in PKD patients with multiple comorbidities the median survival time decreases to the same level with other patients with multiple comorbidities. We have clarified this in the manuscript, page 12, lines 170 and 186.*

Overall, I think this version has better readability. But I think it would be helpful to go over the manuscript again for some minor grammatical revisions.

*Authors’ reply:*

*The manuscript has now been revised and amended in line with the recommendations of the professional language examiner. We hope the text is clearer now.*

Reviewer #2:

Authors have made significant improvement to raised questions. Can the authors comment in conclusions and elaborate how this study will be useful and have implication in changing the current clinical practice.

*Authors’ reply:*

*Thank you for this comment. Many prognostic models have been made to assess the survival of patients starting kidney replacement therapy. However, these models do not take into account the difference in impact of comorbidities on survival in different kidney diagnoses. If you use these models in clinical practice, it may lead to a wrong assessment in some patients and could have an influence on the decision how patient is treated. We have added a sentence on this in the Discussion, page 15, line 247.*
